# The Effect of Non-equispaced Sampling Instants, Sub-period Record Epochs, and Timebase Gain on the Information Content of Discretized Replicas of Periodic Signals

**DOI:** 10.6028/jres.117.005

**Published:** 2012-02-02

**Authors:** N. G. Paulter

**Affiliations:** National Institute of Standards and Technology, Gaithersburg, MD 20899

**Keywords:** digitized replica, equivalent-time sampler, impulse-like waveforms, periodic signals, real-time oscilloscope, sampling instants, step-like waveforms, timebase gain, transient digitizer, waveform recorder

## Abstract

The effect of non-equispaced sampling instants and timebase gain on the information content of the discretized replica of a periodic signal is examined. The effect of the duration of record epochs that are not equal to an integer number of signal periods on the information content of the discretized replica is also explored. A general model describing sampled and windowed data is provided and compared to related models developed by other researchers.

## 1. Introduction

Discrete-time sampling of signals is the method used today to obtain or acquire a digitized waveform or replica of the signal. However, the time discretization process may introduce both amplitude and timing errors into the digitized replica because of the non-ideal behavior of the sampling instrument’s timebase electronics and/or improperly selected sampling parameters. In this paper, we will focus on the timing errors. These errors may be deterministic errors, which are caused by timebase gain errors and non-equispaced sampling instants, and stochastic errors, which are caused by trigger jitter and noise on the trigger signal. Consequently, the effects of sampling on the discretized replica must be understood to assess the information quality of those data. In this paper, the effect of non-equispaced sampling instants and timebase gain (expansion or contraction) on the accuracy of the temporal and spectral information contained in the digitized replica of a repetitive signal is examined. In addition, because record epochs are frequently used in waveform acquisition that are not equal to the signal period, the effect of sub-period epochs on the information content of the digitized replica relative to that of the signal is examined.

The effects of timebase error in sampling have been examined in part by a variety of different researchers [1–11]. Crochiere and Rabiner [1] provide a review of interpolation and decimation of discretized replicas, which is relevant to understanding the effect of timebase gain errors on the spectrum of the replica. Papoulis performs a sampling error analysis that includes the effects of record truncation and aliasing, but not the effects of non-equispaced sampling instants [2]. A description of aliasing can also be found in most texts concerned with signal processing [12]. Aliasing can be avoided, in most cases, if the data sampling rate is fast enough to capture all level (or amplitude) transitions in the signal (see Sec. 2). In some cases, however, aliasing may occur if the signal has not settled to its initial value before the waveform is terminated at the end of the epoch. However, under certain conditions, these abrupt waveform terminations may be dealt with effectively [13–16]. Jenq has written a series of papers dealing with the effect of non-equispaced sampling instants on the discrete spectrum of the digitized replica [3–5] where his focus was on the periodicity of timebase errors. The effects of jitter and trigger signal noise on the digitized replica will not be considered here because they may be equivalently described as a low-pass filter [17] and dealt with accordingly. Amplitude variations that are not caused by the timebase are also not addressed here.

The purpose of this paper is to establish a model that accurately describes the digitized replica of a signal that is acquired by discrete-time sampling of that signal. This model is compared to those previously developed and then used to demonstrate consistency among all the models discussed. We will be primarily concerned with periodic or repetitive signals but will point at application to waveforms of single events (such as those acquired with transient digitizers or oscilloscopes set to trigger on a single event). The discrete spectra of the time-domain waveforms will be used for the comparison of the different models because the spectra allow a comparison of both magnitude and phase. The time-domain replicas are not compared because this comparison would be limited to certain waveform parameters (such as transition duration, settling time, etc.) and the selection of the parameter is dependent on the type of signal being measured.

In Sec. 2, four different models are presented and compared to models previously developed by other researchers. The four models are of increasing complexity and represent different ways of interpreting the discretized replica. These different models represent not only different ways of interpreting a measurement process, but also different measurement processes, such as real-time and equivalent-time sampling. The reason for presenting the different models is to show that they all produce a consistent description of the measurement result. An examination of the errors caused by non-equispaced sampling instants is given in Sec. 3.

The application for which this work is pulse signal measurement and analysis, where the pulse may be either step-like (the replica of the windowed signal has different steady-state levels on either side of the level transition) or impulse-like (the replica of the signal has the same steady-state level on either side of the pulse). Specifically, the concern is with the information fidelity of waveforms having epochs exceeding 10 s and that also require high sampling rates, > 10^9^ samples/s, to capture the fast transients occurring in these waveforms. These waveforms often describe the output of electro-shock weapons (ESW). This analysis has broad applicability because step-like and impulse-like pulses not only describe ESW signals, they are the basis signal transmission in information, communication, and computer technologies. Furthermore, the spectrum of these pulses can be computed without introducing artifacts: the spectrum of an impulse-like waveform can be computed directly and that for a step-like waveform can be computed after a modification to accommodate for the record truncation discontinuity [16]. It will be assumed that the signal has been sampled properly so that the waveform spectrum does not exhibit aliasing effects and that the record truncation problem, if it exists, has been remedied. Typically the instruments that are used to acquire the digitized replicas exhibit timebase errors and amplitude noise. The amplitude noise of the signal is not considered here other than to say that signal noise will limit the amount of amplitude improvement that can be observed or resolved in a non-equispaced-corrected replica compared to an uncorrected replica. We will not address correcting a waveform for non-equispaced sampling instants because this would require the development of an uncertainty analysis which is beyond the scope of this paper.

## 2. Models of the Sampled Signal

The measurement of a continuous-time signal with modern equivalent-time or real-time sampling instrumentation yields an amplitude- and time-discretized replica of that signal. Errors in the time-discretization process will affect the accuracy by which the discretized replica represents the input signal. The replica, *f_n_*, can be described by:

(1) $$f_{n}=f\left(t\right)\delta \left(t-n\alpha \tau_{d}-\Delta_{n}\right);n=0,1,\ldots ,N-1$$

where *f*(*t*) is the continuous-time signal, *δ* is the Kronecker delta function (*δ* (*t* = 0) = 1, *δ* (*t* ≠ 0) = 0), *ϑ_d_* is the ideal equispaced interval between successive samplings, *∀* is the timebase gain coefficient (ideally *α* = 1), *)_n_* are the deterministic corrections that represent the deviations of the actual time intervals from *∀ϑ_d_*, and *N* is the number of acquired data used to represent the signal. There may be *N* unique values of *)_n_* for any *N*-point waveform. The *)_n_* are reproducible for a given epoch and delay setting of the trigger but may be unique for each unique epoch or delay setting. Amplitude noise and jitter (where it will be assumed the jitter is random and normally distributed) will not be considered here. However, the effect of jitter from a finite number of averages will contribute to the uncertainty in *)_n_* and these uncertainties should be propagated in an error analysis of any method used to correct for timebase errors.

Four different models describing the digitized replicas are now presented and compared to each other and to other models developed previously by other researchers, which will show consistency among the model results. These models provide different insight into the construction of the waveform as well as its spectrum. The effect of an observation window (or of windowing) on the spectrum of *f*(*t*) will be considered in Sec. 3.

### 2.1 Model 1

**Fig. 1:**
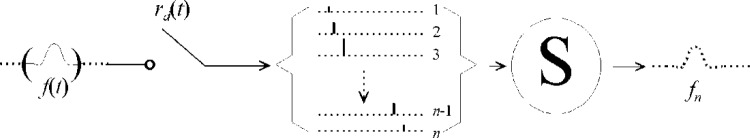
Functional diagram of Model 1. A repetitive signal, *f*(*t*), is sampled at intervals defined by *r_d_*(*t_n_*) to yield a series of subsequences, each with one nonzero value. These subsequences are then summed to yield the sampled waveform, *f_n_*.

The most common model of the discrete-time sampling process is to consider the waveform as the summation of many (*n*) sequences, where each sequence has only one nonzero value [18]. In this model (see Fig. 1), signal discretization is accomplished through a repetitive sampling function, *r_d_*(*t_n_*), where

(2) $$\begin{array}{l} r_{d}\left(t_{n}\right)=\sum_{n=-\infty }^{\infty }\delta \left(t-n\alpha \tau_{d}+\Delta n\right),\\ t_{n}=n\alpha \tau_{d}+\Delta n, \end{array}$$

and *∀ϑ_d_* is the period between sampling instants. For an equivalent-time sampler, *n* is incremented by one only after each trigger event that the sampler is ready to accept. The signal is sampled at the instants given by *r_d_*(*t_n_*), and this sampling provides an average signal value for each sampling instant. The waveform is then constructed by placing these average signal values at their corresponding instants in time.

The digitized replica can be written as:

(3) $$f_{n}=f\left(t\right)r_{d}\left(t_{n}\right).$$

The sampling occurrence function, *r_d_*(*t_n_*), behaves like a mask to allow only those time instants, the *t_n_*, at which *f*(*t*) will be recorded to give *f_n_*. The discrete spectrum of *f_n_*, *F_k_*, is denoted by:

(4) $$\begin{array}{l} F_{k}=\frac{1}{N}\sum_{n=0}^{N-1}\int_{t=0}^{\infty }f\left(t\right)\delta \left(t-n\alpha \tau_{d}+\Delta_{n}\right)e^{-j2\pi \frac{kt}{N\alpha \tau_{d}}}dt\\ \;=\frac{1}{N}\sum_{n=0}^{N-1}f_{n}e^{{}^{-j2\pi \frac{k\left(n\alpha \tau_{d}-\Delta_{n}\right)}{N\alpha \tau_{d}}}}, \end{array}$$

where *f_n_ = f*(*n ∀ϑ_d_+)_n_*) and it is assumed in this model that the signal period is equal to the waveform epoch. The angular frequency is equal to *2π/ϑ_p_* where *ϑ_p_ = N∀ϑ_d_.* The second line in (4) is a result of the integrand in (4) being nonzero only when *t = n ∀ϑ_d_+)_n_.* Equation (4) is identical to the results of Papoulis [18] with appropriate substitutions. Model 1 is very simple and, although it provides the correct solution, does not provide any insight into how *f*(*t*) is created, the effect of sampling on the replica, synchronization requirements, and how spectrum discretization comes about. However, Model 1 is commonly used to describe the discretized replica and, as will be shown later, is accurate except for a scale factor.

### 2.2. Model 2

**Fig. 2:**
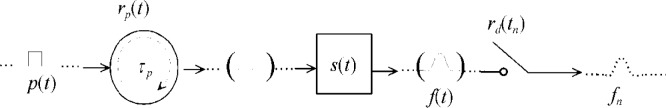
Functional diagram of Model 2. The pulse output is repeated every *τ_p_* intervals and then filtered by *s*(*t*), which represents the impulse response of the measurement instrument. This signal is then sampled as defined by *r_d_*(*t_n_*) to yield the sampled waveform *f_n_*.

Model 2, shown in Fig. 2, which is similar to [1] but more complete than (4) is to explicitly show how *f*(*t*) is generated.

In Fig. 2, the *p*(*t*) is the input pulse, *s*(*t*) is the sampler impulse response or sampler function, and *r_p_*(*t*) is the input signal repetition function which can be written as:

(5) $$r_{p}\left(t\right)=\sum_{k=-\infty }^{\infty }\delta \left(t-k\tau_{p}\right),$$

where *ϑ*_p_ is the signal period. We will assume here that *ϑ_p_* is constant. Examples of *p*(*t*) and *s*(*t*) are as follows. The input signal, *p*(*t*), can be obtained from an arbitrary pulse generator where *r_p_*(*t*) is the programmable repetition rate. The *p*(*t*) may also be a time-varying repetitive current or voltage signal that is the result of the interaction of a transducer with some physical event. The *s*(*t*) is the sampling aperture and is fixed by the sampling instrument.

The spectrum of the *f_n_* described by model 2 is given by:

(6) $$\begin{array}{l} F_{k}=\int_{t=0}^{\infty }\int_{\tau =0}^{\infty }\int_{T=0}^{\infty }p\left[t-\left(\tau -T\right)\right]s\left(\tau -T\right)\sum_{k=-\infty }^{\infty }\delta \left(T-k\tau_{p}\right)\sum_{n=0}^{N-1}\delta \left(t-T-n\alpha \tau_{d}-\Delta n\right)e^{-j\omega t}dTd\tau dt\\ \;=\int_{t=0}^{\infty }\int_{T=0}^{\infty }f\left(t-T\right)\sum_{k=-\infty }^{\infty }\delta \left(T-k\tau_{p}\right)\sum_{n=0}^{N-1}\delta \left(t-T-n\alpha \tau_{d}-\Delta n\right)e^{-j\omega \left(t-T\right)}e^{-j\omega T}dTdt\\ \;=\frac{2\pi }{\tau_{p}}\int_{t=0}^{\infty }\sum_{k=-\infty }^{\infty }\delta \left(\omega -k\omega_{p}\right)\sum_{n=0}^{N-1}f\left(t-T\right)\delta \left(t-T-n\alpha \tau_{d}-\Delta n\right)e^{-j\omega \left(t-T\right)}e^{-j\omega k\tau_{p}}dt\\ \;=\frac{2\pi }{N\alpha \tau_{d}}\int_{t=0}^{\infty }\sum_{n=0}^{N-1}f\left(t\right)\delta \left(t-n\alpha \tau_{d}-\Delta n\right)e^{-j2\pi \frac{kt}{\tau_{p}}}dt\\ \;=\frac{1}{N\alpha \tau_{d}}\sum_{n=0}^{N-1}f_{n}e^{-j2\pi \frac{n\alpha \tau_{d}+\Delta_{n}}{N\alpha \tau_{d}}}. \end{array}$$

Equation (6) shows explicitly how spectrum discretization arises whereas (4) did not. The first line shows that the replica is the result of a convolution of *p*(*t*) with *r_p_*(*t*) (defined by the summation over *k*), followed by a convolution with *s*(*t*), and then a multiplication by *r_d_*(*t_n_*) (defined by the summation over *n*). Line 2 is obtained from line 1 by performing the convolution of *s*(*t*) with *p*(*t*) to *get f*(*t*), this was not shown in Model 1. Knowing how *f*(*t*) is created is also helpful in understanding its discrete spectrum. Line 3 is obtained by performing the Fourier transform of *r_p_*(*t*) and line 4 is obtained by considering only the frequencies allowed by *r_p_*(*t*), namely those frequencies for which *ω = kω_p_ = 2πk/ϑ_p_*, and by doing a change of variables (“*t*-*T*” is replaced by an arbitrary variable “*Z*” which is not shown, and then “*Z*” is subsequently replaced by “*t*”). Line 5 (the last line) is obtained by considering only the time instants allowed by the kronecker-delta in line 4, that is, when *t = n ∀ϑ_d_+)_n_*, and then substituting *ϑ_p_ = N∀ϑ_d_.* The constant “2*π*” in the numerator is eliminated because the fourier transform of a product of two time-domain signals is the convolution of their spectra divided by *2π* [19]. It is assumed in (6) that *)_n_* is unbiased, that is, *∀* is chosen so that

(7) $$\sum_{n=0}^{N-1}\Delta_{n}=0$$

and *|)_n_*| *< ∀ϑ_d_* for all *n.*

Model 2 is more complete than Model 1 because it shows how spectrum discretization arises and how the pulse and sampler interact to form the measured signal *f*(*t*). However, because the data is described to be contained in only one sequence, Model 2 still does not accurately represent an equivalent-time sampler. However, Model 2 does describe a non-averaging real-time acquisition of a repetitive signal. If *r_p_*(*t*) is removed from the model, then this would describe a single-sweep real-time acquisition of a transient (or single-event) signal.

### 2.3 Model 3

**Fig. 3:**
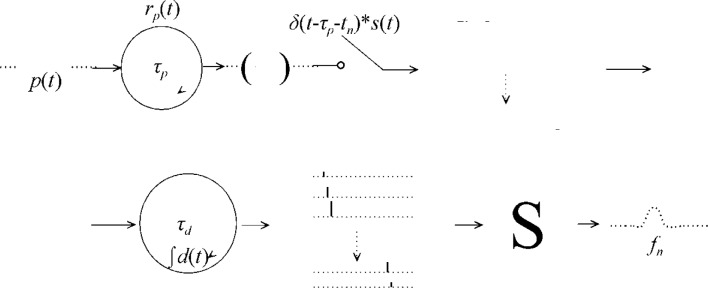
Functional diagram of Model 3. The pulse output is repeated every *τ_p_* intervals, ideally sampled at instants given by (*t*-*τ_p_*-*t_n_*), and filtered by *s*(*t*) to yield a series of subsignals each representing the *p*(*t*) at the given instant. These subsignals are subsequently integrated at intervals given by *τ_d_* to yield a series of subsequences, each of which has only one nonzero element. The subsequences are then summed to yield the sample waveform, *f_n_*. The asterisk indicates a convolution process.

Model 2 is similar to Model 1 but follows more closely to the analysis of Jenq [3]. Model 3 is more representative of the equivalent-time sampling process than Models 1 or 2. Model 3 assumes there are *N* subsequences each consisting of *N* entries and all *N* spectra of the *N* subsequences are summed. This is similar to Model 1 except that here all interactions leading *to f_n_* are now considered (see Fig. 3). Model 3 is different from Model 2 in the interpretation of the sequence *f_n_*: Model 2 describes *f_n_* as one sequence of *N* nonzero terms whereas Model 3 describes *f_n_* as *N* subsequences and each subsequence has only one nonzero term. Whereas Jenq allowed for many nonzero entries for a given subsequence, only one nonzero entry per subsequence is allowed here. Jenq’s allowance provides for periodicity within the subsequences that he used to examine the effect of the periodicity of timebase errors on the spectrum of the replica. For an equispaced reconstruction of a non-equispaced sampled replica, this restriction is not necessary. Each subsequence in this analysis will have a unique equispaced sampling interval, *ϑ_d_,n = ∀ϑ_d_-)_n_*, and a unique location, *δ*(*t-T-t_n_*), where its only nonzero component can be found. For the case of one nonzero entry per subsequence, this model description is the same as Jenq’s [3].

The discrete spectrum of the *f_n_* of Model 3 may be described by:

(8) $$\begin{array}{l} F_{k}=\sum_{n=0}^{N-1}\left(\int_{t=0}^{\infty }\int_{\tau =0}^{\infty }\int_{T=0}^{\infty }p\left(t-\tau \right)s\left(\tau -T\right)\delta \left(t-T-t_{n}\right)\sum_{m=-\infty }^{\infty }\delta \left(T-k\tau_{p}\right)dTd\tau \sum_{m=-\infty }^{\infty }\delta \left(t-T-m\tau_{d,n}\right)e^{-j\omega t}dt\right)\\ \;=\left(\sum_{n=0}^{N-1}\int_{t=0}^{\infty }\int_{T=0}^{\infty }f\left(t-T\right)\delta \left(t-T-t_{n}\right)\sum_{k=-\infty }^{\infty }\delta \left(T-k\tau_{p}\right)\sum_{m=-\infty }^{\infty }\delta \left(t-T-m\tau_{d,n}\right)e^{-j\omega \left(t-T\right)}e^{-j\omega T}dTdt\right)\\ \;=\frac{2\pi }{\tau_{p}}\sum_{n=0}^{N-1}\left[\left(\sum_{k=-\infty }^{\infty }\delta (\omega -k\omega_{p})\right)\int_{t=0}^{\infty }f\left(t-T\right)\delta \left(t-T-t_{n}\right)\sum_{m=-\infty }^{\infty }\delta \left(t-T-m\tau_{d,n}\right)e^{-j\omega \left(t-T\right)}dt\right]\\ \;=\frac{2\pi }{\tau_{p}}\sum_{n=0}^{N-1}\left[f_{n}e^{-jk\omega_{p}t_{n}}*\frac{1}{2\pi }\sum_{m=-\infty }^{\infty }\delta \left(\omega -m\omega_{d,n}\right)\right]\\ \;=\frac{1}{\tau_{p}}\sum_{n=0}^{N-1}f_{n}e^{-jk\omega_{p}t_{n}}\sum_{m=-\infty }^{\infty }\delta \left(\omega -m\omega_{d,n}\right)\\ \;=\frac{1}{N\alpha \tau_{d}}\sum_{n=0}^{N-1}f_{n}e^{-j2\pi \frac{n\alpha \tau_{d}-\Delta_{n}}{N\alpha \tau_{d}}}. \end{array}$$

The first line shows that the replica is the result of a convolution of *p*(*t*) with *s*(*t*), followed by a convolution with *r_p_*(*t*) (defined by the summation over *k*), and then a multiplication by *r_d_*(*t_n_*) (defined by the summation over *n*). In line 1, the *n* subscript in *ϑ_d,n_* implies that each subsequence may have a different nominal sampling interval. The second line is obtained by performing the convolution of *p*(*t*) with *s*(*t*) and the third line by performing the Fourier transform of *r_p_*(*t*); the same that was done for Model 2. In the third line, the term *exp*(-*jωkϑ_p_*) is not shown because the discretization of *ω* causes this term to equal one for specific values of *k* and is zero otherwise (same as was done for Model 2). In line 4, *ω_d,n_* was substituted by *2π*/*ϑ_d,n_.* The fourth line results from the fact that the Fourier transform of a product of two functions in the time-domain is a convolution of their spectra in the frequency-domain and this correctly represents the equivalent-time sampling process. Elimination of the constant “2*π*” was done as in Model 2. Also, in the fourth line, only that part of the spectrum of *f*(*t*) allowed by *δ*(*ω-kω_p_*) is shown, that is, the spectrum is discretized. The fifth line is obtained because the convolution of the infinite sum with a discrete-frequency constant value is a multiplication of the sum by that value. The last line is obtained by performing the summation over *m* and forcing the sum to normalize to one. The sum must equal one because the sum does not add or subtract energy from the signal. The *ϑ_d,n_* is expanded in the last line to show and *∀* and *)_n_.* A comparison to Jenq’s results [3] shows that this result is slightly different from his. Although Jenq assumed that the delay between the first nonzero values of each subsequence was constant, we have not assumed that here. If we do make that assumption, however, then *)_n_* = 0 and the argument of the exponent in line 6 of (8) becomes *j*2*πkn/N* where *2πk/N* is equivalent to Jenq’s “ω.”

### 2.4 Model 4

Model 4 is the most representative of an equivalent-time sampler. Here, *s*(*t*) is applied to *p*(*t*) after the appropriate delay, which is *ϑ_p_* + *nϑ_d_*, where *n* is the equivalent-time sampling instant. The discrete spectrum for this model of an equivalent-time sampler is:

(9) Fk=∑n=0N−1(∫t=0∞∫τ=0∞∫T=0∞∫T=0∞p(t−T−τ)s(τ−T)δ(t−T−T−tn)∑m=−∞∞δ(T−kτp)∑m=−∞∞δ(τ−mτs)e−jωtdTdTdτdt)=(∑n=0N−1∫t=0∞∫T=0∞∫T=0∞f(t−T−T)δ(t−T−T−tn)∑k=−∞∞δ(T−kτp)∑m=−∞∞δ(T−mτs)e−jω(t−T)e−jωTdTdTdt)=2πτp∑n=0N−1[(∑k=−∞∞δ(ω−kωp))∫t=0∞∫T=0∞f(t−T−T)δ(t−T−T−tn)∑k=−∞∞δ(T−kτp)∑m=−∞∞δ(T−mτs)e−jω(t−T−T)e−jωTdTdt]=2πτp∑n=0N−1[∫t=0∞f(t−T−T)∑k=−∞∞δ(ω−kωp)∑m=−∞∞δ(ω−mωs)δ(t−T−T−tn)e−jω(t−T−T)dt]=2πτp∑n=0N−1[∫t=0∞f(t−T−T)δ(t−T−T−tn)e−jω(t−T−T)dt]=1τp∑n=0N−1f(tn)e−jkωptnNατd1Nατd∑n=0N−1fne−j2πknατd−ΔnNατd.

The description of the derivation of the solution for Model 4 is very similar to that of Model 3. There are a couple of differences. The primary difference between Model 3 and Model 4 is that Model 3 uses a truncated set of an infinitely sampled series whereas model 4 uses a limited sampled set, the latter more realistically representing an equivalent-time sampler. Also, in Model 4, the requirement that the pulse repetition rate and the sampling rate are synchronized is shown explicitly (as exhibited by the summations over *k* and *m* in line 3).

The four different models that were presented herein for interpreting the discretized replica all yield identical results, except for a scale factor, and these models are consistent with those presented by other researchers. Models 2, 3, and 4 accurately describe real-time and equivalent time sampling processes. Therefore, we can perform an examination of the effect of non-equispaced sampling on the replica and to correct, if we elect to do so, for non-equispaced sampling without worrying about the interpretation of the measurement process. The correction process is beyond the scope of this paper, as previously stated.

### 2.5 Pulse and Sampler Synchronization, Sampling Intervals, and Spectrum Discretization

It is worth expanding on the topic of pulse and sampler synchronization to understand the requirements for obtaining a sensible waveform. To show pulse-sampler synchronization, *f*(*t*) may be written symbolically as a convolution of the sampler response function, *s*(*t*); the sampler repetition function, *r_s_*(*t*); the pulse output function, *p*(*t*); and the pulse repetition function, *r_p_*(*t*); that is,

(10) $$f\left(t\right)=s\left(t\right)*r_{s}\left(t\right)*p\left(t\right)*r_{p}\left(t\right),$$

where the “*” indicates a convolution. The *r_s_*(*t*) describes the time interval between successive sampling events. The convolution process shown in (10) becomes a multiplication of spectra in the frequency domain, that is:

(11) $$F\left(\omega \right)=P\left(\omega \right)R_{p}\left(\omega \right)S\left(\omega \right)R_{s}\left(\omega \right),$$

where *P*(*ω*), *S*(*ω*), *R_p_*(*ω*), and *R_s_*(*ω*) are the spectra of *p*(*t*), *s*(*t*), *r_p_*(*t*), and *r_s_*(*t*). Equation (11) can be simplified because the products *P*(*ω*)*R_p_*(*ω*) and *S*(*ω*)*R_s_*(*ω*) are discrete, that is,

(12) $$S\left(\omega \right)R_{s}\left(\omega \right)=S\left(\omega \right)\sum_{m=-\infty }^{\infty }\delta \left(\omega -m\omega_{s}\right),$$

(13) $$P\left(\omega \right)R_{p}\left(\omega \right)=P\left(\omega \right)\sum_{L=-\infty }^{\infty }\delta \left(\omega -L\omega_{p}\right),$$

where *ω_s_* = 2*π*/*ϑ_s_* and *ω_p_* = 2*π*/*ϑ_p_*. From (11), (12), and (13), we can see that the frequency components of *F*(*ω*) with nonzero magnitudes are found only at integer multiples of *ω_p_* or *ω_s_*: therefore, *F*(*ω*) is discrete. Moreover, (11), (12), and (13) show that *T_p_* and *T_s_* must be related by a rational number for *F*(*ω*) to contain useful data, that is, the signal and the sampling instants must be synchronized, which agrees with observation. The effect of windowing the signal is to artificially change *r_p_*(*t*) and this will be discussed in Sec. 3.

**Fig. 5:**
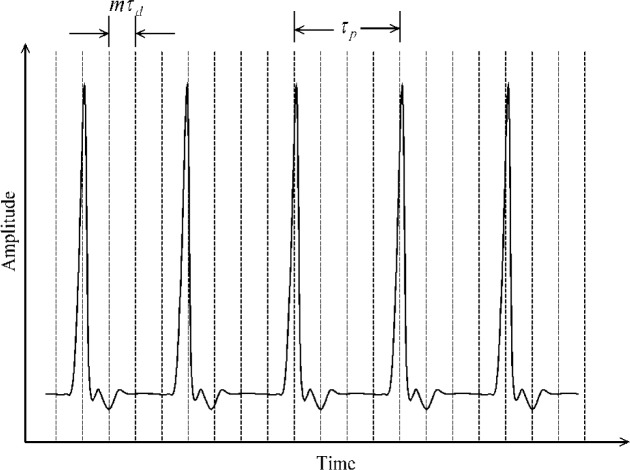
Periodically repetitive waveform showing waveform period, *ϑ_p_*, and an integer number, *m*, of sampling intervals, *ϑ_d_*.

**Fig. 6:**
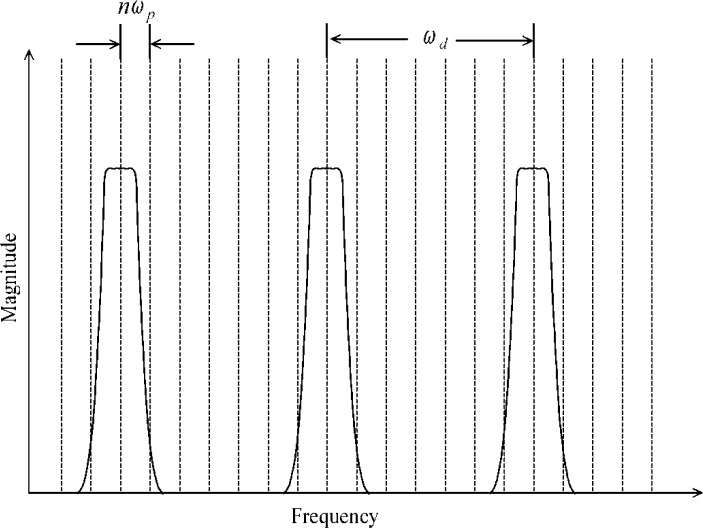
The spectrum of the waveform in Fig. 1 showing the frequency intervals, *ω_d_* and *ω_p_*, caused by the sampling interval, *ϑ_d_*, and waveform period, *ϑ_p_*.

Equation (10) and the subsequent text regard continuous-time measured signals, that is, there is no sampling. For a time-discretized replica, there is additional discretization in the spectrum caused by *r_d_*(*t_n_*). The *r_d_*(*t_n_*) describes the delay in the occurrence of subsequent sampling events relative to a common instant in the measured signal. The product of *r_d_*(*t*) and *f*(*t*) yields *f_n_* (see Eq. 1), which has a discrete spectrum. To understand how *r_d_*(*t_n_*) affects *f_n_*, (see Fig. 5) the spectrum of *r_d_*(*t_n_*) must be examined. The spectrum of the error-free *r_d_*(*t_n_*) is:

(14) $$R_{d}\left(\omega \right)=\sum_{m=-\infty }^{\infty }\delta \left(\omega -m\omega_{d}\right),$$

where *ω_d_* = 2*π*/*ϑ_d_*. Recall that a product of two functions (*r_d_*(*t_n_*) and *f*(*t*)) in the time-domain becomes a convolution of their corresponding spectra in the frequency domain. Consequently, the allowed spectral values of the *F_k_*, which is the discrete Fourier transform of *f_n_*, are found at the nonzero values of *F*(*ω*)*R_d_(*ω*). The frequency spacing of this convolution is given by the frequency spacing of *F*(*ω*) (which has frequency spacings of *ω_p_* and *ω_s_*) and that of the sampling repetition function, which has a spacing of *ω_p_*. Furthermore, the convolution *F*(*ω*)*R_d_(*ω*) is also periodically repeated every *ω_d_* (See Fig. 6). Therefore, if the product *P*(*ω*)*S*(*ω*) extends beyond *∀ω_d_*/2, then frequency information from adjacent spectral periods will overlap. In Fig. 6, these spectral periods do not overlap. This overlap of adjacent spectral periods causes an error, called aliasing, that affects both *F_k_* and *f_n_*. To prevent or minimize aliasing, *ϑ_d_* should be decreased thereby increasing *ω_d_*.

If any of *ϑ_p_*, *ϑ_d_*, and *ϑ_s_* show some random behavior, then the distributions associated with those random behaviors will effectively appear as jitter. Consequently, these distributions will individually or collectively act to low-pass filter *f*(*t*) and can be dealt with accordingly [17].

## 3. Record Errors

In this section we will examine errors caused by non-equispaced sampling and sub-period (windowed) epochs. For sub-period epochs, impulse-like and step-like waveforms will be considered separately. Several investigators have examined and measured the errors in the timebase of waveform recorders [20–23] and developed methods to correct for these errors [24].

### 3.1 Sub-period Epochs, Impulse-like Waveforms

If the data are obtained over an interval not equal to *ϑ_p_* (a sub-period epoch) then (5) must be rewritten to reflect that change, namely:

(15) $$r_{p}\left(t\right)=\sum_{k=-\infty }^{\infty }\delta \left(t-k\left[\tau_{p}-\Delta_{p}\right]\right),$$

where *ϑ_p_* -*)_p_* is the interval over which data are taken. The values of *)_p_* may be determined using methods to measure timebase errors [20–23]. In (5), the signal period is *ϑ_p_* and *ϑ_p_* causes the spectrum of the ideal replica to be discretized with a discrete frequency interval of *ω_p_* = *2π*/*ϑ_p_*, as described in Sec. 2.5 and for the model given in (9). If we acquire the data over an epoch *ϑ_p_* -*)_p_*, then the discrete frequency intervals become $\omega_{p}^{\prime }=2\pi /{\left(\vartheta_{p}-\right)}_{p})$*.* We can determine which spectral components of the windowed replica are valid, that is, which spectral components of the windowed replica have counterparts in the ideal replica’s spectrum, by equating the *m*^th^ harmonic of *ω_p_* with the *n*^th^ harmonic of $\omega_{p}^{\prime }$:

(16) $$m\omega_{p}=n\omega_{p}^{\prime }\;or\;\frac{m2\pi }{\tau_{p}}=\frac{n2\pi }{\tau_{p}-\Delta_{p}}.$$

Equation (16) can be rearranged to give:

(17) $$\frac{\Delta_{p}}{\tau_{p}}=1-\frac{n}{m}.$$

Equation (17) can be used to determine which spectral components of the windowed replica have a corresponding component in the spectrum of the ideal replica. For example, if the epoch is 0.9*ϑ_p_* (*)_p_* = 0.1 *ϑ_p_*) then *n/m* = 9/10. This means that every 9th component of the windowed replica’s spectrum is valid because it contains the information from every 10th component of the ideal replica’s spectrum. As another example, let *)_p_* = 0.9*ϑ_p_*. In this case, each component of the windowed replica’s spectrum is valid because it contains information from every 10th component of the ideal replica’s spectrum. Although each component in the spectrum of the windowed replica may not have a corresponding component in the ideal replica, the spectrum of the windowed replica may be interpolated to approximate the spectrum of the ideal waveform and, therefore, the waveform itself. However, resonances, etc. will make this sort of approximation dubious. What is commonly assumed when *)_p_* ≠ 0 is that the pulse is a single event (so that *ϑ_p_***→∞** and then *ω_p_*→0) and that any frequency information contained in the windowed replica is also contained in the signal. This assumption is, of course, wrong. It is important to realize that the imposition of a sub-period epoch is artificial and the resultant discretized replica should not be expected to contain the same spectral information as the signal. However, this error is basically the introduction of false components into the spectrum of the windowed replica of an impulse-like signal and it is possible to determine which of these components are false.

### 3.2 Sub-period Epochs, Step-like Waveforms

For brevity, we will consider equations describing continuous-time signals. However, the result is the same for discrete-time replicas. A sub-period epoch for a step-like waveform can be described as a windowed signal:

(18) $$f^{w}\left(t\right)=f\left(t\right)w\left(t\right),\;w\left(t\right)=\{\begin{array}{ccc} 1 & for & 0\leq t\leq T_{D}\\ 0 & & \mathrm{otherwise} \end{array}$$

where *w*(*t*) is the rectangular windowing function and *T_D_* is the duration of the window, *T_D_* < *ϑ_p_*. The spectrum of *f^w^*(*t*) is:

(19) $$F^{w}\left(\omega \right)=F\left(\omega \right)*W\left(\omega \right),$$

where *W*(*ω*) is given by:

(20) $$\begin{array}{c} W\left(\omega \right)=\int_{-\infty }^{\infty }w\left(t\right)e^{-j\omega t}dt\\ \;=\int_{-T_{D}/2}^{T_{D}/2}e^{-j\omega t}dt\\ \;=\frac{2}{\omega }\sin\left(\omega \frac{T_{D}}{2}\right). \end{array}$$

From (18) we can see that the windowed signal, *f^w^*(*t*), has lost information relative to *f*(*t*) whenever *f*(*t*) *≠* 0 outside the interval 0 ≤ *t ≤ T_D_. In* addition, the spectrum of the windowed signal has been corrupted by the window, see (19) and (20). Therefore, truncating a signal before the signal has time to relax to its initial value causes distortion of the spectral content of the windowed signal relative to the input signal. This distortion is in addition to the introduction of false components, which occurs for windowed impulse-like signals.

### 3.3 Non-equispaced Sampling Instants

The effect of non-equispaced sampling instants on the discretized replica are examined using the following:

(21) $$E_{n}=f_{I,n}-f_{n},$$

where *f_I,n_* is the ideal equispaced replica and *f_n_* is the non-equispaced waveform. The frequency domain error, using the discrete Fourier transform of (21) and using the model in (9), is:

(22) $$E_{k}=\sum_{n=0}^{N-1}\left(f_{I,n}-f_{n}\right)e^{-\frac{j2\pi k}{N}\left(\frac{n\alpha \tau_{d}-\Delta_{n}}{\tau_{d}}\right)}.$$

Clearly, if *f_I,n_* = *f_n_*, then the error is zero for *E_n_* for the given *n*. However, for *E_k_* = 0 for any *k* requires *f_I,n_* = *f_n_* for all *n* and if this is the case, then *E_k_* = 0 for all *k*.

Another way to look at the effect of non-equispaced sampling instants is to examine the power of the error waveform or its spectrum. The power of the error waveform is:

(23) $$P_{err,time}=\sum_{n=0}^{N-1}E_{n}^{2}=\sum_{n=0}^{N-1}{\left(f_{I,n}-f_{n}\right)}^{2}.$$

Equation (23) presupposes that the *f_n_* are at the correct sampling instants and, therefore, has no mechanism for incorporating the case of *Δn* ≠ 0. But as we know, *Δn ≠* 0, so we need a way of examining their effects on *f_n_* and *F_k_.* The spectrum of (23) may provide more information because *Δn* can be shown. Rewriting (23) for the frequency domain gives:

(24) $$P_{err,freq}=\sum_{k=0}^{N-1}E_{k}^{2}=\sum_{k=0}^{N-1}\left(F_{I,k}-F_{k}\right){\left(F_{I,k}-F_{k}\right)}^{*},$$

where the asterisk indicates the complex conjugate. Using the discrete Fourier transforms of *f_I,n_* and *f_n_*, and considering only one index of *k* for brevity, we get:

(25) $$\begin{array}{l} P_{err,k}=\frac{1}{N}\{\sum_{n=0}^{N-1}f_{n}^{2}+\sum_{n=0}^{N-1}f_{I,n}^{2}-2\sum_{n=0}^{N-1}f_{n}f_{I,n}\cos\left(\frac{2\pi k\Delta_{n}}{N\tau_{d}}\right)\\ \;+\sum_{\begin{array}{c} n,m=0\\ n\neq m \end{array}}^{N-1}f_{n}f_{m}\cos\left(\frac{2\pi k}{N\tau_{d}}\left[\left(n-m\right)\tau_{d}+\left(\Delta_{n}-\Delta_{m}\right)\right]\right)\\ \;+\sum_{\begin{array}{c} n,m=0\\ n\neq m \end{array}}^{N-1}f_{I,n}f_{I,m}\cos\left(\frac{2\pi k}{N}\left[n-m\right]\right)\\ \;-2\sum_{\begin{array}{c} n,m=0\\ n\neq m \end{array}}^{N-1}f_{n}f_{I,m}\cos\left(\frac{2\pi k}{N\tau_{d}}\left[\left(n-m\right)\tau_{d}+\Delta_{n}\right]\right)\} \end{array}$$

The sums in the second and third lines each equals zero because the equal magnitude terms have opposite polarity and cancel for the summation over both *n* and *m*. That is,

$$f_{n}f_{m}\cos\left(\frac{2\pi k}{N\tau_{d}}\left[\left(n-m\right)\tau_{d}+\left(\Delta_{n}-\Delta_{m}\right)\right]\right)+f_{m}f_{n}\cos\left(\frac{2\pi k}{N\tau_{d}}\left[\left(m-n\right)\tau_{d}+\left(\Delta_{m}-\Delta_{n}\right)\right]\right)=0$$

for all *n* and *m* where *n* ≠ *m.* Under most normal situations, *f_I,n_* ≈ *f_n_*, so that the sum over *n* and *m* in the fourth line will be approximately zero and can be ignored, which gives for *P_err,_k*:

(26) $$P_{err,k}≅\frac{1}{N}\left\{\sum_{n=0}^{N-1}f_{n}^{2}+\sum_{n=0}^{N-1}f_{I,n}^{2}-2\sum_{n=0}^{N-1}f_{n}f_{I,n}\cos\left(\frac{2\pi k\Delta_{n}}{N\tau_{d}}\right)\right\}.$$

Summing over the frequencies, gives:

(27) $$P_{err,k}≅\sum_{n=0}^{N-1}f_{n}^{2}+\sum_{n=0}^{N-1}f_{I,n}^{2}-\frac{2}{N}\sum_{k=0}^{N-1}\sum_{n=0}^{N-1}f_{n}f_{I,n}\cos\left(\frac{2\pi k\Delta_{n}}{N\tau_{d}}\right).$$

Equation 27 shows the effect of non-equispaced sampling on the power spectrum error. If *)_n_* = 0 for all *n*, (27) gives the same result as that shown in (23). *If f_I,n_ = f_n_*, (23) will equal zero but (27) will not equal zero unless *)_n_* = 0 for all *n*, which is due to the inclusion of non-equispaced sampling intervals in (27). The *)_n_* can be positive or negative depending on whether the actual sampling instant is greater than or less than the ideal sampling instant. To get an idea of the effect of *)_n_ on P_errk_*, note that (27) will be sinusoidal due to *)_n_*, and will have the greatest values when *)_n_* = ± *τ_d_*/2 and the smallest values when *)_n_ = −τ_d_*, 0, and *τ_d_.*

### 3.4 Timebase Gain

Timebase gain, causing either expansion or contraction, does not affect the waveform or its spectrum parameters other than for a scale factor for temporal or frequency parameters. Timebase gain can be determined using timebase measurement methods previously developed [20–23]. For example, if *α* ≠ 1, and it is assumed that *α* = 1, then temporal parameters, such as transition duration, pulse duration, waveform period, delays, etc., will be in error by a factor, α*_f_*, which can be given by:

$$\alpha_{f}=\frac{\alpha }{\alpha_{0}},$$

where *α*_0_ is the ideal or actual timebase gain. The timebase scale and values of temporal parameters can be multiplied by 1/*α_f_* to correct their values. The frequency spacing in the spectrum, which is given by $\frac{1}{N\alpha \tau_{d}}$, will also need to be corrected, which is accomplished by multiplying the value of this spacing by *α_f_*.

## 4. Conclusion

Models describing the measurement processes used to acquire time-discretized replicas of continuous-time signals have been presented and the models were shown to yield consistent descriptions of the acquired waveforms. These models were also shown to be equivalent to the models previously presented by other researchers. The effect of sub-period recording epochs was also examined which showed that, for impulse-like pulse signals, some of the spectral content of the replica will accurately represent the spectral content of the pulse. For step-like pulse data, however, the spectral content of the replica will not represent that of the input signal. The error caused by non-equispaced sampling is not easy to calculate because the ideal replica is typically not available. However, trends in these errors can be estimated.

## Figures and Tables

**Fig. 4:**
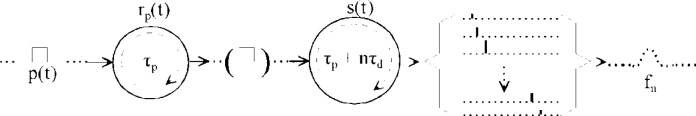
Functional diagram of Model 4. The pulse output is repeated every *τ_p_* intervals to yield a repetitive signal that is periodically sampled by *s*(*t*) at intervals given by *τ_p_* + *nτ_d_*. This results in a series of subsequences, each with only one nonzero value. These subsequences are then summed to yield the sample waveform, *f_n_*.
